# Deciphering lipid dysregulation in ALS: from mechanisms to translational medicine

**DOI:** 10.1186/s40035-022-00322-0

**Published:** 2022-11-07

**Authors:** Ira Agrawal, Yong Shan Lim, Shi-Yan Ng, Shuo-Chien Ling

**Affiliations:** 1 Department of Physiology, Yong Loo Lin School of Medicine, National University of Singapore, Tahir Foundation Building, MD1, 16-03-H, 12 Science Drive 2, Singapore, 117549 Singapore; 2 Healthy Longevity Translational Research Programme, Yong Loo Lin School of Medicine, National University of Singapore, Singapore, Singapore; 3 Institute of Molecular and Cell Biology, A*STAR Research Entities, Singapore, Singapore; 4 Program in Neuroscience and Behavior Disorders, Duke–National University of Singapore Medical School, Singapore, Singapore

**Keywords:** Amyotrophic lateral sclerosis, Sphingolipids, Triglycerides, Phospholipids, Cholesterol esters, Fatty acids, Ceramides, Arachidonic acid, Lysophosphatidylcholine, Eicosanoids

## Abstract

Lipids, defined by low solubility in water and high solubility in nonpolar solvents, can be classified into fatty acids, glycerolipids, glycerophospholipids, sphingolipids, and sterols. Lipids not only regulate integrity and fluidity of biological membranes, but also serve as energy storage and bioactive molecules for signaling. Causal mutations in *SPTLC1* (serine palmitoyltransferase long chain subunit 1) gene within the lipogenic pathway have been identified in amyotrophic lateral sclerosis (ALS), a paralytic and fatal motor neuron disease. Furthermore, lipid dysmetabolism within the central nervous system and circulation is associated with ALS. Here, we aim to delineate the diverse roles of different lipid classes and understand how lipid dysmetabolism may contribute to ALS pathogenesis. Among the different lipids, accumulation of ceramides, arachidonic acid, and lysophosphatidylcholine is commonly emerging  as detrimental to motor neurons. We end with exploring the potential ALS therapeutics by reducing these toxic lipids.

## Background

Amyotrophic lateral sclerosis (ALS) is a neurodegenerative disease characterized by progressive loss of motor neurons in the motor cortex and the spinal cord. Correspondingly, patients show progressive muscle weakness, loss of muscle control and voluntary movement, slurred speech, paralysis, and respiratory failure [1, 2]. The relentless progression of ALS is fast, where the patients succumb typically within 1–5 years after disease onset [1, 2]. At present, this fatal disease has no cure. Current available treatments, riluzole and edaravone, slow symptom progression with life extension for a few months [2, 3]. The exact etiology for ALS remains enigmatic; about 90% cases have unknown causes (termed as sporadic ALS) and 10% are hereditary cases (known as familial ALS). For familial ALS, at least 42 genes, including *SOD1* (superoxide dismutase 1), *C9ORF72* (chromosome 9 open reading frame 72)*, TARDBP* (TAR DNA-binding protein) and *FUS,* have been identified to be causal [4]. Clinically, familial and sporadic ALS are indistinguishable, with familial ALS showing earlier disease onset [5], suggesting that these ALS-causing mutations may predispose the disease risk by triggering similar underlying pathogenic mechanisms in sporadic ALS. As such, genetic causes for familial ALS are used to generate disease models for mechanistic investigations as well as translational studies [6–9]. Furthermore, based on genome-wide association studies (GWAS) and epidemiological studies, it is likely that ALS arise from a complex pathogenic process involving multiple genetic factors and/or gene-environment interactions [10]. Indeed, numerous pathways and mechanisms are known to contribute to ALS, including irregular RNA processing, impaired protein homeostasis, elevated oxidative stress, excitotoxicity, deficits in cytoskeleton organization and intracellular transport, and mitochondrial dysfunction [7, 8, 11].

Besides the complex pathogenic process, early diagnosis of ALS remains challenging, and can take up to one year due to confounding disorders, dependency on medical history, as well as clinical examination by electromyography, advanced neuroimaging, and nerve conduction studies [2, 12]. Disease progression is measured by the ALS functional rating scale-revised (ALSFRS-R), which is dependent on subjective evaluation of patients' life quality and may lack sensitivity [2]. To objectively track disease progression, reliable and readily accessible molecular biomarkers, such as those from patients’ blood, that offer speedy, sensitive, and accurate diagnosis and prognosis are necessary [2]. One of the approaches to identifying these biomarkers is through investigating ALS pathophysiology and mechanisms [1, 2, 12]. For example, pathological transactive response DNA-binding protein 43 (TDP-43) (encoded by *TARDBP*) and neurofilament aggregations are found in ALS motor neurons. Neurofilament in blood and cerebrospinal fluid (CSF) and TDP-43 in the CSF have been proposed as diagnostic biomarkers [13, 14], where their elevated levels correlate with disease severity and progression [13–15].

Recently, causal mutations in *SPTLC1,* which encodes serine palmitoyltransferase (SPT) long-chain base subunit 1 that is important for sphingolipid biosynthesis, have been identified in ALS [16, 17], suggesting that deficits in lipid metabolism could drive ALS pathogenesis. Elevated levels of arachidonic acid, a polyunsaturated fatty acid (PUFA), have been shown to contribute to motor neuron dysfunction and death in ALS [18]. Consistent with the notion of lipid-mediated toxicity, lipid dysregulation in the central nervous system (CNS) and circulation in ALS patients have been shown to be clinically associated with disease severity, functional decline and survival [19–23]. Studies have also demonstrated the benefits of a high-caloric diet, especially a lipid-rich diet, in increasing survival in an ALS mouse model [24] and slowing disease progression in ALS patients [25–27]. Patients with elevated serum levels of triglycerides (TGs) [22] and low-density lipoprotein (LDL)/high-density lipoprotein (HDL) ratio [21] have extended survival by nearly a year. In addition, we and others have also shown that TDP-43, the pathological hallmark and one of the causal genes for ALS, regulates sterol regulatory element-binding protein 2 (SREBP2)-mediated cholesterol metabolism [28, 29]. Taken together, these observations indicate that lipid dysmetabolism is an integral part of ALS pathogenesis, and thus, lipids could serve as biomarkers and therapeutic targets.

Recent advances in analytical chemistry techniques allow for large-scale identification of metabolites and lipids, giving rise to metabolomics and lipidomics research [30–32]. These quantitative measurements in patient samples of serum, CSF and spinal cord, patient-derived induced pluripotent stem cell (iPSC)-based cellular models, and ALS genetics-based mouse models, have generated a wealth of data [2, 32]. Here, we review on these studies from a systematic and critical perspective. The main aims are to (i) provide mechanistic insight into how lipid dysmetabolism may contribute to ALS pathogenesis, and (ii) probe the utilization of lipids as biomarkers and therapeutics. Using lipidomic and metabolomic studies as well as cellular and animal models as a guide, we attempt to delineate the diverse roles of different lipid classes in ALS. Among them, accumulation of ceramides, arachidonic acid, and lysophosphatidylcholine (lysoPC) is a consistent theme that appears to be detrimental to motor neurons [18, 33–35]. In contrast, increased levels of glucosylceramides and activation of sphingosine-1-phosphate (S1P)-mediated signaling may be protective in ALS [36–39]. We end with exploring the potential ALS therapeutics by reducing these toxic lipids. We hope the review will pave the way for future work exploring the potential applications of lipids as biomarkers and novel targets for ALS therapeutics.

## Brief introduction of lipid classes and functions in biological systems

**Fig. 1:**
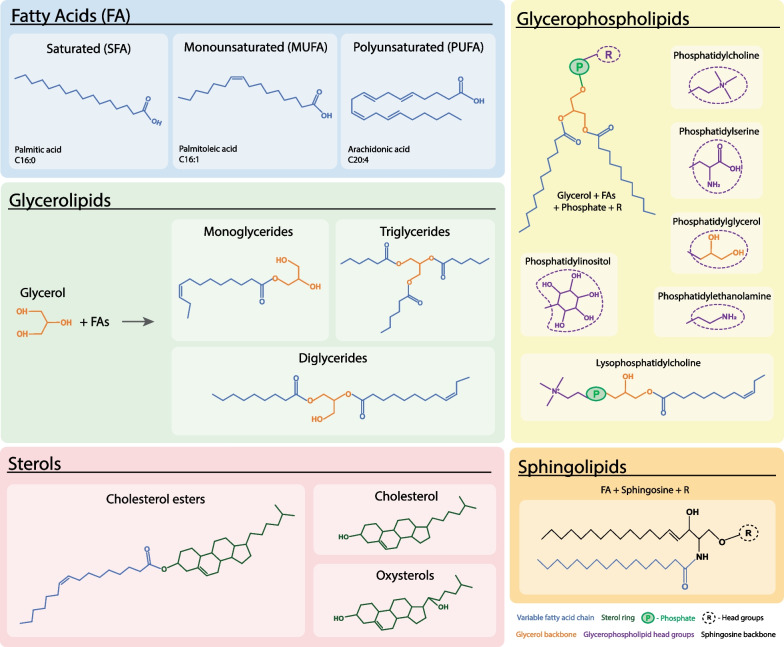
Structural classification of lipids in biological systems. Presented here are the broad lipid classes of fatty acids, glycerolipids, glycerophospholipids, sphingolipids and sterols, and their main sub-classes, along with representative structures. Fatty acids (shown in blue) form the core of most lipid classes and are highly variable with differing chain lengths and saturation. Fatty acids with no double bonds, one double bond and multiple double bonds are further classified into saturated fatty acids, monounsaturated fatty acids, and polyunsaturated fatty acids, respectively. Glycerolipids are formed on addition of fatty acids to a glycerol backbone, while glycerophospholipids have additional phosphate and head groups added. Sphingolipids contain a sphingosine backbone attached with a fatty acid chain. Sterols are tetracyclic ring structures. Glycerol backbone, sphingosine backbone, phosphate group, glycerophospholipid head groups and sterol rings are colored orange, black, light green, purple, and dark green, respectively

**Fig. 2:**
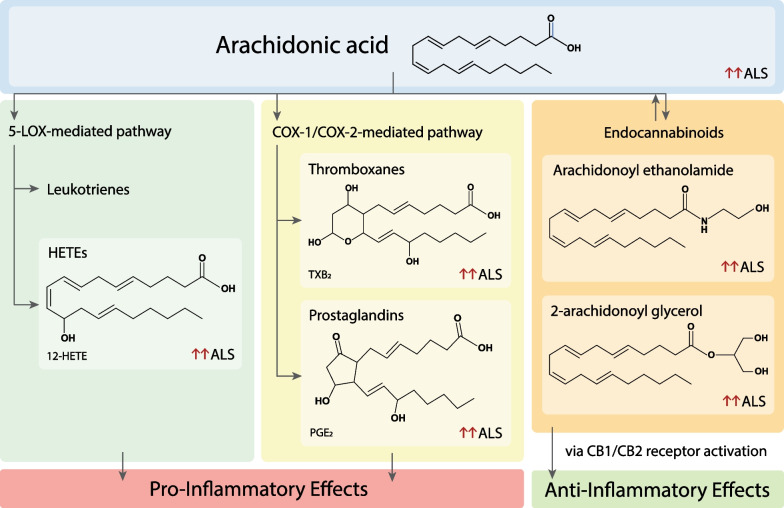
Representative structures of arachidonic acid-derived eicosanoid classes and associated inflammatory effects. Eicosanoids are derived from arachidonic acids, via either the 5-LOX-mediated pathway or COX-1/COX-2-mediated pathway. The 5-LOX-mediated pathway gives rise to eicosanoids such as leukotrienes and hydroxy-eicosatetraenoic acids, whereas the COX-1/COX-2-mediated pathway produces thromboxanes and prostaglandins. These eicosanoids are elevated in ALS disease states and exert pro-inflammatory effects. Endocannabinoids are synthesized reversibly from arachidonic acids. Arachidonoyl ethanolamide (AEA) and 2-arachidonoyl glycerol (2-AG) are endocannabinoids that are upregulated in ALS disease states. Binding and subsequent activation of AEA and 2-AG to CB1/CB2 receptors activates anti-inflammatory response

Lipids are a diverse and complex group of water-insoluble organic compounds with tens of thousands of known species. Lipids in biological systems can be classified into five broad classes: fatty acids, sphingolipids, glycerolipids, glycerophospholipids, and sterols [30]. Figure 1 presents these individual lipid classes, their main sub-classes, and representative structures. Fatty acids form the building blocks of these complex lipid classes, except sterols that are characterised by a steroid backbone [30]. Fatty acids are carboxylic acids with aliphatic carbon chains, defined by the length and saturation of the carbon chain. Specifically, they are classified by the length of their carbon chain (C_n_) into short- (≤ C5), medium- (C6–12), long- (C13–21), and very long-chain fatty acids (≥ C22), and by the number and position of the carbon–carbon double bonds into saturated (no double bonds, C_n_:0) and unsaturated fatty acids (presence of double bonds) [40–42]. Due to their diversity, the unsaturated fatty acids are further classified into monounsaturated (one double bond, C_n_:1) and polyunsaturated fatty acids (> 1 double bond, C_n_:D). Most natural fatty acids have even numbers of carbon, usually between C14 and C24 [40–42]. Additionally, oxidized fatty acid derivatives form a large superfamily of bioactive lipids called eicosanoids, which consist of various sub-groups such as prostaglandins, thromboxanes, hydroxyeicosatetraenoic acids, leukotrienes and endocannobinoids [41–44] (Fig. 2).

Fatty acids are rarely found in free state in nature, and are often stored as TGs, also known as triacylglycerols [40–42]. TGs are part of the glyceride lipid class, which consists of one, two or three fatty acids bound to a glycerol via an ester linkage to form monoglycerides, diglycerides and TGs, respectively [40–42]. Triacylglycerols are the most common form of glycerolipids and can be hydrolyzed back to fatty acids when required by the body, thereby serving as an energy reserve [40–42]. Glycerophospholipids are phosphorylated diglycerides characterized by their various head groups. The head groups include choline, ethanolamine, serine, inositol, or glycerol, thus forming phosphatidylcholine (PC), phosphatidylethanolamine (PE), phosphatidylserine (PS), phosphatidylinositol and phosphatidylglycerol, respectively [40–42]. Lysophospholipids contain a single fatty acid chain with a polar head group [40–42].

**Fig. 3:**
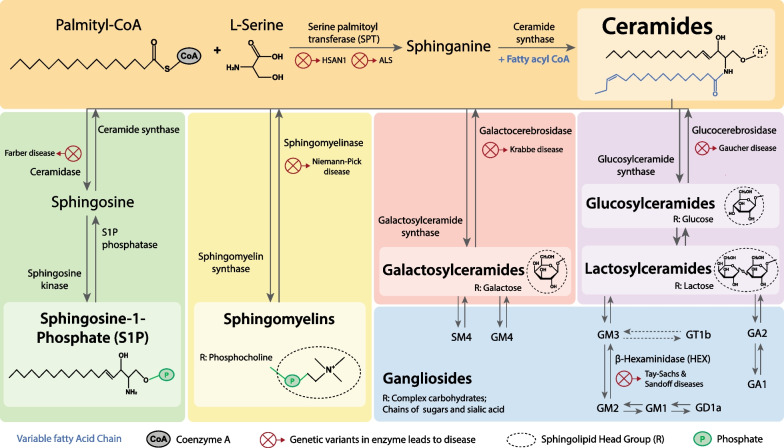
Sphingolipid classes, metabolism, and associated diseases. Ceramides are the basic structural unit of sphingolipids, and can be synthesized de novo from palmitoyl coA, *L*-serine and fatty acyl coA with variable length (shown in blue), or from the breakdown of the more complex sphingolipid classes - glucosylceramides, galactosylceramides, lactosylceramides, gangliosides and sphingomyelin. Sphingosine-1-phosphate is the breakdown product of ceramide and is an active signaling molecule. Genetic variants of many of the key enzymes causing sphingolipid accumulation are causal for various neurodegenerative and metabolic diseases, including ALS and have been specified in the figure

Sphingolipids represent another lipid class, whose backbone is sphingosine rather than glycerol as in the case of glycerolipids and phospholipids [40–42]. Sphingolipids are synthesized by addition of a sphingosine to fatty acids by an amide linkage to form ceramides. Ceramides could be either esterified with a phosphorylcholine to form sphingomyelin (SM) or glycosylated with one or more sugars to form glycosphingolipids [45]. Glycosphingolipids are further classified into cerebrosides and gangliosides based on the complexity of added sugars. Cerebrosides use a single sugar moiety like glucose (glucosylceramide), galactose (galactosylceramide) or lactose (lactosylceramide). Gangliosides use complex carbohydrates as their head groups and are formed by the consecutive addition of galactose and sialic acid to glucosylceramide, galactosylceramide or lactosylceramide [46]. Examples of gangliosides are GM1, GM2, GM3, GD1a and the GQ1 series (see Fig. 3).

The last major lipid class is sterols, which are characterized by a hydrophilic hydroxylated tetracyclic sterane structure [40–42]. They are primarily synthesized via the mevalonic acid pathway and can be found free or as esters with hydrophobic fatty acid chains [47]. The lipid classes are interrelated and can change form from one class to another via various chemical reactions.

The large diversity of lipids allows for varying properties and roles. Known roles of lipids are metabolic substrates and energy reserves, structural components of membranes, and signaling molecules [48, 49]. As mentioned, TGs serve as an energy reserve and can be broken down via β-oxidation in the mitochondria. Lipids are integral structural components of cellular membranes, with phospholipids forming the characteristic membrane lipid bilayer and sterols and sphingolipids creating hydrophobic lipid rafts within it. The lipid composition in the membrane dictates membrane fluidity and permeability, which in turn affects the capacity of cell membrane to exchange substrates and proteins, as well as for signal transduction. Specialized lipid domains, known as lipid rafts, that are enriched with glycosphingolipids, cholesterol and protein receptors, influence signaling processes, receptor trafficking and neurotransmitter transport [50, 51]. Lipids themselves could act as signaling molecules and are involved in multiple signaling pathways, such as inflammation, oxidative stress, neurogenesis, cell–cell communication, neurotransmitter transport, and glucose homeostasis, and as ligands by acting on various families of nuclear receptors such as peroxisome proliferator-activator receptors and liver X receptor (LXR)/retinoid X receptor (RXR) to influence transcription [52–54].

Above we only highlight and summarize the essential knowledge for the purpose of understanding this review. Interested readers are encouraged to explore more comprehensive and in-depth reviews for lipid structures and functions [49, 55–57].

## Overview of ALS lipidomic studies

To understand how lipid dysmetabolism may contribute to ALS pathogenesis, we have summarized the pertinent ALS metabolomic and lipidomic findings of broad lipid classes from human patient samples and animal models in Tables 1, 2, 3. A lipid class is marked to have higher or lower levels if the majority of significantly differentially expressed species in the lipid class are upregulated or downregulated in ALS patients as compared to healthy controls; otherwise, it is marked as mixed expression. This section discusses the ALS lipidomic signatures by tissue type, and potential lipid biomarkers identified. We will discuss each lipid class in more detail in the later sections.

**Table 1:** Summary of levels of major lipid classes in blood samples of human ALS patients from metabolomic and lipidomic studies

Tissue source	Blood				
References	Lawton et al. [34]	Goutman et al. [60]	Sol et al. [61]	FernÁndez-Eulate et al. [63]	Area-Gomez et al. [62]
Number of ALS patients	172	125	23	20	40
Tissue details	Plasma	Plasma	Plasma	Fasting serum	Fasting serum
Experiment	Metabolomics	Metabolomics	Lipidomics	Lipidomics	Lipidomics
Species detected	367	1051	1018	416	~ 500
Differential species	32	303	122	28	23
*Sphingolipids*					
Sphingomyelins	Higher	Higher	Higher	Higher	Mixed
Ceramides	n.d.	Higher	Mixed	Higher	Higher
Glucosylceramides	n.d.	Higher	n.a.	n.d.	n.s.
Galactosylceramides	n.d.	n.d.	Lower	n.d.	n.s.
Lactosylceramides	n.d.	Higher	Higher	n.d.	Higher
Globosides	n.d.	n.d.	n.a.	n.d.	n.s.
Gangliosides	n.d.	n.d.	n.a.	n.d.	Higher
*Glycerolipids*					
Diglycerides	n.d.	Higher	Mixed	n.s.	Higher
Triglycerides	n.d.	n.s.	Higher	Higher	Higher
*Glycerophospholipids*					
Phosphatidylcholine	n.s.	Mixed	Higher	Mixed	Lower
Phosphatidylethanolamine	Higher	n.s.	Higher	n.s.	Mixed
Phosphatidylinositol	Higher	n.s.	Higher	n.s.	n.s.
Phosphatidylserine	n.d.	n.s.	n.a.	n.d.	Lower
Phosphatidylglycerol	n.d.	n.s.	Mixed	n.d.	n.s.
Lysophospholipids	Higher	Higher	Higher	Higher	Lower
*Fatty acids*					
Saturated fatty acids	Higher	Higher	n.a.	n.s.	n.s.
Monounsaturated FA	Higher	Higher	Higher	Mixed	Higher
Polyunsaturated FA	Higher	Higher	Higher	Lower	Higher
Arachidonate (20:4)	Higher	Higher	n.a.	n.s.	n.a.
*Sterols*					
Cholesterol esters	n.d.	n.d.	n.a.	n.s.	Higher
Cholesterol	Higher	n.d.	n.d.	n.d.	Higher

*n.s.*, not significantly different; *n.d.*, not detected; *n.a.*, unknown/information not available

**Table 2:** Summary of levels of major lipid classes in CSF and spinal cords of human ALS patients from metabolomic and lipidomic studies

Tissue source	CSF		Spinal cord		
References	Blasco et al. [65]	Sol et al. [61]	Cuttler et al. [33]	Dodge et al. [36, 209]	Ramírez- Nuñez et al. [70]
Number of ALS patients	40	23	9	6 male	4
Tissue details	CSF	CSF	Lumbar spinal cord	Spinal cord gray matter	Nuclei of lumbar spinal cord
Experiment	Lipidomics	Lipidomics	Targeted	Targeted	Lipidomics
Species detected	200	843	4 lipid classes	9 lipid classes	n.a.
Differential species	19	17	n.a.	n.a.	151
*Sphingolipids*					
Sphingomyelins	Higher	n.a.	Higher	Higher	n.a.
Ceramides	n.d.	n.a.	Higher	Higher	Mixed
Glucosylceramides	Higher	n.a.	n.d.	Higher	Lower
Galactosylceramides	n.d.	n.a.	n.d.	Higher	n.a.
Lactosylceramides	n.d.	n.a.	n.d.	Higher	Higher
Globosides	n.d.	n.a.	n.d.	Higher	n.a.
Gangliosides	n.d.	n.a.	n.d.	Higher	n.a.
*Glycerolipids*					
Diglycerides	n.d.	Lower	n.d.	n.d.	Mixed
Triglycerides	Lower	Lower	n.d.	Higher	Mixed
*Glycerophospholipids*					
Phosphatidylcholine	Higher	n.a.	n.s.	n.d.	Higher
Phosphatidylethanolamine	n.s.	n.a.	n.s.	n.d.	Higher
Phosphatidylinositol	n.s.	n.a.	n.s.	n.d.	n.s.
Phosphatidylserine	n.d.	n.a.	n.s.	n.d.	Mixed
Phosphatidylglycerol	n.d.	n.a.	n.s.	n.d.	Mixed
Lysophospholipids	Higher	n.a.	n.d.	Higher	Higher
*Fatty acids*					
Saturated fatty acids	n.d.	n.a.	n.d.	n.d.	n.d.
Monounsaturated FA	n.d.	n.a.	n.d.	n.d.	n.d.
Polyunsaturated FA	n.d.	n.a.	n.d.	n.d.	n.d.
Arachidonate (20:4)	n.d.	n.a.	n.d.	n.d.	n.d.
*Sterols*					
Cholesterol Esters	n.s.	n.a.	Higher	Higher	n.d.
Cholesterol	n.d.	n.d.	n.d.	n.s.	n.d.

*n.s.*, not significantly different; *n.d.*, not detected; *n.a.*, unknown/information not available

**Table 3:** Summary of levels of major lipid classes in ALS animal models from metabolomic and lipidomic studies

Tissue	Spinal cord				
References	Cutler et al. [33]	Dodge et al. [36]	Henriques et al. [67]	Chaves-Filho et al. [69]	Burg et al. [68]
Organism	Mouse	Mouse	Mouse	Rat	Mouse
ALS-like genotype	SOD1-G93A	SOD1-G93A	SOD1-G86R	SOD1-G93A	Human FUS overexpression
Tissue details	Lower spinal cord	Spinal cord	Spinal cord	Spinal cord	Spinal cord
Type	Targeted	Targeted	Lipidomics	Lipidomics	Lipidomics
Species detected	4 lipid classes	9 lipid classes	1599 lipids	406 lipids	897 lipids
Differential species	n.a.	n.a.	46 lipids	23 lipids	106 lipids
*Sphingolipids*					
Sphingomyelins	Higher	n.s.	Mixed	n.s.	Lower
Ceramides	Higher	Mixed	Lower	Higher	Mixed
Glucosylceramides	n.d.	Higher	Lower	n.s.	n.d.
Galactosylceramides	n.d.	Lower	n.a.	n.d.	n.d.
Lactosylceramides	n.d.	n.s.n.s.	n.a.	n.d.	n.d.
Globosides	n.d.	Lower	n.a.	n.d.	n.d.
Gangliosides	n.d.	Higher	Higher	n.d.	n.d.
*Glycerolipids*					
Diglycerides	n.d.	n.d.	n.a.	n.s.	Mixed
Triglycerides	n.d.	n.d.	Lower	n.s.	n.s.
*Glycerophospholipids*	–	–			
Phosphatidylcholine	n.s.	n.d.	Mixed	n.s.	Higher
Phosphatidylethanolamine	n.s.	n.d.	Mixed	n.s.	Mixed
Phosphatidylinositol	n.s.	n.d.	Higher	n.s.	Lower
Phosphatidylserine	n.s.	n.d.	n.s.	n.s.	Mixed
Phosphatidylglycerol	n.s.	n.d.	n.s.	n.s.	Higher
Lysophospholipids	n.d.	n.d.	n.s.	n.s.	Higher
*Fatty acids*					
Saturated fatty acids	n.d.	n.d.	n.a.	n.s.	n.d.
Monounsaturated FA	n.d.	n.d.	n.a.	n.s.	n.d.
Polyunsaturated FA	n.d.	n.d.	n.a.	n.s.	n.d.
Arachidonate (20:4)	n.d.	n.d.	n.a.	n.s.	n.d.
*Sterols*					
Cholesterol esters	Higher	n.d.	n.a.	Higher	Higher
Cholesterol	n.d.	n.s.	n.s.	n.d.	n.d.

*n.s.*, not significantly different; *n.d.*, not detected; *n.a.*, unknown/information not available

### Plasma of ALS patients

Given the non-invasive nature, blood sampling from patients  is an ideal source for biomarkers. Not surprisingly, many of the ALS metabolite studies have investigated blood samples from patients (Table 1). Due to the blood–brain barrier (BBB) and blood-spinal cord barrier (BSCB), which limit the exchange of metabolites between the CNS and the peripheral blood, there are two obvious caveats to be considered. The first is whether the changes in blood lipids are a reflection of or a response to the changes in the CNS or vice versa? The second is how leaky BBB and BSCB during ALS progression [58, 59] contribute to the changes in blood and brain lipids? The latter point would also inevitably introduce greater variations among different studies.

An early metabolomics study of plasma samples from 172 ALS patients identified 367 metabolites. Among them, a panel of 32 metabolites could distinguish ALS samples from neurological disease mimic samples and healthy controls with 90% specificity [34]. The panel of 32 metabolites contained 11 highly expressed  lipids, including saturated fatty acids, arachidonic acid, SM, cholesterol, and cortisone. The levels of the panel lipids correlated with disease severity tested by the ALS functional rating scale (ALSFRS-R score) [34]. Subsequent studies using plasma samples with a wider detection range show similar trends of higher expression levels of a large number of SM and fatty acid species [60–62]. Goutman et al*.* additionally identified higher levels of ceramides, glucosylceramides, lactosylceramides, diacylglycerides and lysophospholipids [60].

A two-year longitudinal lipidomic study investigated serum samples from patients with ALS  or primary lateral sclerosis (PLS), a motor neuron disease that targets upper motor neurons. The two diseases have similar lipid profiles, but could be distinguished by dysregulation of glycerophospholipids in ALS that is not seen in PLS [62]. ALS patient serum also has elevated expression of specific cholesterol esters, ceramides and SM species that correlates with disease progression, which was not observed in the PLS patients [62]. However, another lipidomic study using fasting serum could not accurately discriminate ALS patients from healthy controls despite changes in lipid profiles [63]. Nevertheless, it identified four lipids that have consistently higher expression in ALS patient serum, including two monounsaturated fatty acids (24:1 and 14:1), a TG (TG 51:4) and a  SM (SM 36:2) [63].

Plasma samples from pre-symptomatic individuals, who developed ALS within five years of sample collection, showed mild dysregulation of glycerolipids, cholesterol esters, PC, and SM. However, no significant changes or signatures could be identified to predict diseased individuals [64].

### CSF of ALS patients

The other biofluid of interest in ALS studies is the CSF, which may contain lipids released by damaged cells under the diseased state. The ALS CSF lipidomic signature is distinct from the plasma, with only 19 and 17 differential lipids identified in two studies [61, 65] (Table 2). The most discriminant molecule in the CSF is the increased PC 36:4 [65], which is also observed in the brains of SOD1-G93A ALS mice [66]. SM and glucosylceramides are also observed to be elevated in the CSF [65]. Interestingly, TG levels are reduced in the CSF of ALS patients, while the plasma of the same patients show an increased level of TGs [61] (see “Glycerolipids in ALS” section for details).

### Spinal cords of ALS patients and ALS mouse models

Degeneration of spinal cord motor neurons is a characteristic of ALS. Therefore, spinal cord is expected to be the site of high metabolite dysregulation. Lipidomic studies from spinal cords of ALS patients and ALS mouse models are summarized in Tables 2 and 3. Targeted mass spectrometry studies of spinal cord tissues from postmortem ALS patients showed elevated levels of cholesterol esters and a range of sphingolipids including SM, ceramides, glucosylceramide, galactosylceramide, lactosylceramide, globosides and gangliosides [33, 36]. In addition, the spinal cord gray matter has elevated levels of TGs and lysoPC [35]. Some of these changes have also been observed in ALS mouse models. The spinal cords of SOD1-G93A mouse model show elevated levels of specific ceramides, glucosylceramide, gangliosides and cholesterol esters [33, 36], whereas the SOD1-G86R mice have elevated levels of gangliosides and phosphatidylinositol and lower levels of ceramides and glucosylceramides [67]. The ALS FUS mouse model (which overexpresses wild-type human FUS) has elevated levels of cholesterol esters and specific ceramides, and dysregulation of phospholipids, including lower levels of cardiolipin [68]. Downregulation of cardiolipins has also been observed in SOD1-G86R rat spinal cords, along with a nearly six-fold increase in cholesterol esters [69].

Lipid composition changes in the isolated nuclei of the spinal cord from ALS patients include altered expression of diglycerides, TGs, plasmalogens and glycerophospholipids, which are known to be major components of the nuclear membrane [70]. This suggests changes in lipid composition of the nuclear membrane and nucleoplasm in the ALS neurons.

### ALS cellular models and other tissues, including muscles, from ALS animal models

ALS patient fibroblasts and iPSC-derived neurons have also been studied in terms of lipid profiling. ALS patient fibroblasts display many mitochondrial defects similar to those found in ALS motor neurons, and have been used to study mitochondrial metabolism and to discover biomarkers for ALS [71, 72]. The lipidome of skin fibroblasts from ALS patients has elevated levels of SM, ceramides, and phospholipids [73]. Among them, PC 36:4 is also observed to be highly discriminatory in CSF of ALS patients [61] and mouse models [66].

A recent study analyzed ALS patient iPSC-derived neurons using multi-omics approaches, including genomics, proteomics, and metabolomics. Higher levels of arachidonic acids and phospholipids such as PE, PS, phosphatidylglycerol, and lysophospholipids, are observed in spinal cord motor neuron cultures compared to ocular motor neuron cultures derived from human ALS-iPSCs cell lines [18]. Among the lipid species, the elevated arachidonic acid has been proposed to play a role in the selective vulnerability of spinal cord motor neurons in ALS [18].

Besides spinal cords, lipidomic studies have also been carried out in muscles and motor cortex tissues from SOD1-G86R mice [67] and SOD1-G86R rats [69], respectively. Lipidomic analysis of skeletal muscles from SOD1-G86R mice revealed similar findings as the spinal cord signatures of ALS patients, with increased levels of ceramides and glucosylceramide and dysregulation of phospholipids [67]. It should be noted that progressive loss of neuromuscular junctions (NMJs) is a key pathological feature in ALS [74]. While whether ALS genetic risk factors would intrinsically affect muscle function and degeneration remains to be addressed, muscles and NMJs remain a primary and attractive site for therapeutic intervention [75, 76]. Concerning the motor cortex, SOD1-G86R rats show increased levels of ceramides, glucosylceramide and phospholipids in the motor cortex [69]. However, changes in the lipid composition are mostly associated with age rather than with the disease symptomatic stage [69].

Overall, these lipidomic changes remain largely distinct between various disease samples. This could be due to the differences in disease status, CNS and peripheral systems, affected cell and tissue types, etc. Furthermore, there could be some compensatory mechanisms, such as the regulation of lipid transport, uptake, and utilization. Nevertheless, common themes may be emerging from these studies. In the following, we will discuss lipidomic changes observed in each lipid class and sub-class in detail.

## Fatty acids in ALS

In the CNS, excess fatty acids are stored as lipid droplets primarily in astrocytes, and the lipid droplet formation is increased in response to hypoxia, cellular stress, and exposure to high levels of exogenous free fatty acids. About 20% of the total brain energy needs are provided by ATP generated by fatty acid β-oxidation mainly occurring in astrocytes [77]. Fatty acid oxidation produces more energy as compared to glucose, but also takes up more oxygen resources. Thus, prolonged usage of fatty acid β-oxidation places cells under oxidative stress, leading to the production of harmful reactive-oxygen species. Unlike neurons, astrocytes generate a large number of antioxidant molecules and are also the major site of lipid storage and oxidation in the CNS [78]. It has been proposed that due to the high energy demand and impaired glucose metabolism in ALS, there is a switch to using lipids as an energy source via fatty acid oxidation in both neurons and astrocytes. This switch in energy source may place the system under elevated oxidative stress, thereby contributing to motor neuron death in ALS [23, 79, 80].

Fatty acids are stored as TGs in lipid droplets, and released for utilization under starvation conditions [55, 81]. A recent study showed that C9ORF72, whose hexanucleotide repeat expansion is causal for ALS [82, 83], regulates lipid metabolism under starvation conditions [84]. Specifically, C9ORF72 deletion leads to reduced lipid droplets and increased de novo fatty acid synthesis under starvation conditions, accompanied by upregulation of NOX2. NOX2 is a NADH oxidase that is known to cause oxidative stress and has been shown to be upregulated in ALS patients [85, 86]. This C9ORF72-dependent starvation-related lipid dysmetabolism is mediated by preventing the degradation of coactivator-associated arginine methyltransferase 1 (CARM1). Furthermore, CARM1 upregulation and C9ORF72 reduction are observed in the spinal cords of C9ORF72-linked ALS patients [85, 86]. Taken together, these findings further support the notion that increased fatty acid utilization as an energy source in ALS may lead to excess oxidative stress. However, fatty acids represent more than an energetic support for neural cells. Emerging evidence suggests that fatty acids may play important roles in ALS disease progression.

### Saturated fatty acids

As discussed above, greater fatty acid metabolism triggers increased oxidative stress, leading to production of peroxidated toxic lipids. These peroxidated lipids are released from neurons and taken up by astrocytes, which either break down the lipids or store them as lipid droplets. This neuron-astrocyte coupling of lipid metabolism is protective for neurons [78, 87]. Interestingly, Guttenplan et al*.* demonstrated that neurotoxic reactive astrocytes secrete long-chain saturated free fatty acids that contribute to cell death [88]. Fractionation of conditional media of neurotoxic reactive astrocytes with column chromatography led to identification of apolipoprotein E (APOE)- and apolipoprotein J-containing lipoprotein particles with long-chain saturated free fatty acids, which contribute to the observed toxicity. Consistently, unbiased lipidomics of more than 1500 lipids from 10 classes revealed significant upregulation of long-chain saturated free fatty acids in the conditioned media of these reactive astrocytes. To reduce the production of long-chain fatty acids, Guttenplan and colleagues performed astrocyte-specific knockout (KO) of ELOVL fatty acid elongase 1 (ELOVL1), an enzyme that catalyzes the synthesis of long-chain (> C16) fatty acids. The conditional media from ELOVL1-KO astrocytes  were less toxic than that of wild-type mice, indicating that the long-chain saturated free fatty acids secreted by astrocytes trigger cell death [88]. In the context of ALS, although higher levels of saturated fatty acids have been reported in the plasma of ALS patients [34, 60], it is not known whether there is enhanced expression or activity of astrocytic ELOVL1. Furthermore, whether ALS astrocytes also secrete more long-chain free fatty acids that may be part of astrocyte-induced toxicity in ALS remains to be addressed.

### Unsaturated fatty acids

Unsaturated fatty acids have bends or ‘kinks’ in their structure due to the presence of double bonds (Fig. 1), which disallows the molecules from packing tightly. Thus, most unsaturated fatty acids are liquids at room temperature, which has many biological implications such as maintaining membrane order and fluidity, and the positions of double bonds affect function [89]. Unsaturated fatty acids are sub-grouped by the number of double bonds into monounsaturated fatty acids (MUFA) and polyunsaturated fatty acids (PUFA).

#### MUFA

The conversion of MUFAs to saturated fatty acids, catalyzed by stearoyl-CoA desaturase (SCD), is sensitive to energy metabolism requirements of the body. SCD indices, which are the ratios of palmitoleate to palmitate (16:1/16:0) and oleate to stearate (18:1/18:0), have been shown to associate with lifestyle, diet and body composition [90]. Significantly, increased levels of 16:1 and 18:1, along with increased indices (16:1/16:0 and 18:1/18:0), have been reported in the blood cells of ALS patients [91]. Further, the 16:1 level and 16:1/16:0 ratio were observed to negatively correlate with disease progression as tracked over a 6-month period. By contrast, other markers that are used in the measurement for obesity, such as body mass index or leptin concentration, do not correlate. Patients with higher 16:1/16:0 ratios have significantly improved survival, suggesting that this ratio could be used as a marker for disease prognosis [91]. Interestingly, a MUFA-enriched diet ameliorates disease symptoms and increases the survival rate of ALS SOD1-G93A mouse model [92]. However, higher plasma and serum levels of MUFA have been reported in ALS patients [34, 60–62], and the MUFAs C24:1 and C14:1 are consistently increased in ALS patient serum samples [63]. However, the exact role of MUFAs in ALS remains to be clarified.

#### PUFA

PUFAs have been proposed to mediate motor neuron toxicity in ALS and regulate inflammatory responses, with elevated levels observed in ALS patient plasma [34, 60–62]. Omega-3 (ω-3) and omega-6 (ω-6) fatty acids, i.e., fatty acids with the first double bond from the methyl tail end at the third and sixth carbons, respectively, are of special interest since they are important precursors of various key bio-signaling molecules called eicosanoids. ω-3 fatty acids, such as eicosapentaenoic acid (EPA, 20:5(ω-3)) and docosahexaenoic acid (DHA, 22:6(ω-3)), give rise to anti-inflammatory eicosanoids. By contrast, ω-6 PUFAs, such as linoleic acid (18:2(ω-6)) and arachidonic acid (20:4(ω-6)), give rise to pro-inflammatory eicosanoids [93]. Since mammals are unable to convert ω-6 PUFAs to ω-3 PUFAs, or synthesize PUFAs de novo, tissue levels of these PUFAs and their associated eicosanoids are directly linked to their dietary intake [43, 94]. Dietary supplementation of ω-3 fatty acids  has been shown to offer various benefits in rodents, including reduced neuroinflammation and improved spatial memory [95]. Consistent with the potential beneficial role of ω-3 fatty acids, a longitudinal study using questionnaire data on 995 ALS patients concluded that a lower risk of ALS is associated with a greater intake of ω-3 fatty acids [96]. However, dietary supplementation of EPA accelerates disease progression and shortens the lifespan of SOD1-G93A mice, despite decreased neuroinflammation [97]. Intriguingly, another study in SOD1-G93A mice demonstrated that higher supplementation of ω-6 fatty acids delays disease progression, while dietary supplementation with equal amounts of ω-3 and ω-6 fatty acids accelerates disease progression and death [98]. These studies suggest that a fine balance of ω-3 and ω-6 may need to be maintained, and disruptions of the intake ratio between ω-3 and ω-6 may influence ALS disease progression.

Arachidonic acid (an ω-6 PUFA) and DHA (an ω-3 PUFA) are two major PUFAs present in high concentrations in the CNS. DHA has been reported to be elevated in the brains and spinal cords of ALS patients [99]. Arachidonic acid, which is found at higher levels in plasma of ALS patients, is part of a 32-metabolite panel that is not only discriminatory but also positively correlates with the disease severity [34]. One mechanism to increase arachidonic acid could be the hydrolysis of membrane phospholipids by cytosolic phospholipase A2 (cPLA2). In this regard, the expression and activity of cPLA2 are elevated in the spinal cords of ALS patients [100], as well as in motor neurons of SOD1-G93A mice [101]. Furthermore, the released arachidonic acid may then be metabolized by cyclooxygenases (COXs), lipoxygenases (LOXs) and cytochrome P450 enzymes into multiple biologically active eicosanoids, such as prostaglandins, thromboxanes, prostacyclins, and leukotrienes, which also act as mediators of inflammatory response. Not surprisingly, elevated levels of arachidonic acid have been proposed to contribute to neurotoxicity via elevated neuroinflammation.

### Fatty acid derivatives: eicosanoids

Eicosanoids (*eicosa*-: Greek for “twenty”) are a large and divergent superfamily of oxygenated derivatives of PUFAs. They are potent, but short-lived, cell signaling molecules involved in regulating inflammation, immune response, pain perception, and allergies [43, 94]. They are most frequently derived from arachidonic acid, followed by EPA, and are the mediators of the effects of PUFAs. Arachidonic acid can be metabolized by 5-liopxygenase to generate leukotrienes (LTs), such as 5-hydroperoxy-6,8,11,14 eicosatetraenoic acid (5-HPETE) and subsequently other LTs, or by cyclooxygenases 1 and 2 (COX1, consecutively expressed in most of tissues, and COX2, inducible expression) to generate prostaglandins and thromboxanes [102] (Fig. 2). Arachidonic acid-derived eicosanoids have been studied extensively in ALS and are discussed below.

A recent study by Lee and colleagues provided further evidence that the arachidonic acid pathway contributes to motor neuron dysfunction and death in ALS [18]. Using patient-derived iPSC harboring ALS mutations, Lee and colleagues compared the molecular signatures of derived spinal motor neurons (affected cell type in ALS) and ocular motor neurons (unaffected cell type in ALS). Co-analysis of transcriptomics and metabolomics data identified activation of the arachidonic acid pathway as a common feature of ALS spinal motor neurons. In particular, metabolomic analyses revealed significant down-regulation of a 5-lipoxygenase (5-LOX) inhibitor analog in ALS spinal motor neuron cultures. Indeed, when testing known 5-LOX inhibitors as potential therapeutic agents in ALS iPSC-derived motor neurons, a *Drosophila* model of C9ORF72 ALS and SOD1-G93A mice, the results showed promotion of survival [18].

Profiling of arachidonic acid derivatives show increased levels of LOX-derived metabolites, such as hydroxy-eicosatetraenoic acids (12-hydroxyeicosatetraenoic acid and 15-hydroxyeicosatetraenoic acid), and COX-derived prostaglandins (prostaglandin E2 [PGE_2_] and Prostaglandin D2) and thromboxane B2, in SOD1-G93A mice [103]. Increased levels of PGE_2_ and enzymes involved in its biosynthesis and metabolism have been reported in serum and CSF of ALS patients as well as in spinal cords of SOD1-G93A mice [104–107]. A pilot study in 50 sporadic ALS patients and controls found elevated 15-F2t-isoprostane in urine samples of the ALS patients [108]. The 15-F2t-isoprostane is derived from arachidonic acid via free-radical catalysed mechanisms, and is a well-known oxidative stress marker extensively used in various diseases, including Alzheimer’s disease [109]. A positive correlation has been found between increased urinary concentration of a prostaglandin D2 metabolite, 11,15-dioxo-9-hydroxy-,2,3,4,5-tetranorprostan-1,20-dioic acid, and ALS progression [110]. However, the study was limited to only six ALS patients and controls [110].

Recently, endocannabinoids, also derivatives of PUFAs, have received attention for ALS therapeutics. They are naturally occurring eicosanoid sub-family molecules and act as non-classical retrograde neurotransmitters, which bind and activate the cannabinoid receptors 1 and 2 (CB1 and CB2, respectively) [111]. Activation of cannabinoid receptors in turn activates the anti-glutamatergic and anti-inflammatory responses, which are neuroprotective in nature [44, 112, 113]. Arachidonoyl ethanolamide (AEA) (also called anandamide) and 2-arachidonoyl glycerol (2-AG) are two endocannabinoids abundant in humans [44, 113] (Fig. 2), and both have been reported to be elevated in spinal cords of SOD1-G93A mice [114]. Furthermore, serum concentrations of AEA and 2-AG were found to be elevated and predict ALS in a study of 47 ALS patients and controls [115]. Upregulation of CB2 receptor has been reported in ALS patient spinal cords and motor cortex [116], and in spinal cord of a canine ALS model [116].

## Glycerolipids in ALS

Glycerolipids are neutral lipids and act as precursors to other lipids in the CNS. They are most abundantly found in astrocytes within lipid droplets [55, 81]. Glycerolipids can be phosphorylated to form glycerophospholipids, or hydrolyzed to give rise to fatty acids of various chain lengths. In high energy demand conditions, glycerolipids are quickly depleted to produce fatty acids for energy metabolism. ALS patients with elevated serum TG levels are reported to have prolonged life expectancy, suggesting that serum level of TGs could be used as a prognostic factor [22].

Lipidomic studies have reported higher levels of diglycerides [60, 62] and TGs [61–63] in the sera of ALS patients, of which the TG 51:4 is a reliable discriminant lipid to distinguish patient samples from healthy controls. A longitudinal study with a two-year follow-up after the first measurement, found increased diglyceride and TG levels but decreased monoglyceride levels in ALS patients at later stages of the disease [62]. Interestingly, the diglyceride species containing MUFAs like palmitoleic (16:1) and oleic (18:1) acids are significantly elevated. TGs containing the same MUFAs are also elevated though not significantly. This is consistent with the previously discussed study which reported higher MUFA/saturated fatty acid ratios (18:1/18:0 and 16:1/16:0) in ALS patient blood cells, negatively associated with disease prognosis [91]. Elevated levels of diglycerides, which are precursors to TGs in serum, suggest increased de novo glyceride synthesis, or mobilization from adipose tissue, or both. Additionally, in the plasma, TG 68:12 levels are found to be associated with serum levels of neurofilament, an established neuronal damage marker [61].

Interestingly, two CSF lipidomic studies both reported lower TG levels in ALS patients [61, 65], with TG 16:1/18:1/18:2 being a discriminatory molecule to distinguish between ALS and healthy control samples, while decreased levels of TG 16:0/16:0/18:1 and TG 18:0/16:0/18:1 correlated with improved survival [65]. Furthermore, TG levels of C16 and C18:1 species are increased up to three folds in spinal cords of male ALS patients and are also elevated in spinal cords of SOD1-G93A mice [35]. In mouse spinal cords, TG accumulation increases with disease progression and is predominant in gray matter [35]. However, it remains to be determined if these observations indicate a greater consumption of glycerolipids in the CNS in order to meet the energy demand and if there is a switch to fatty acid oxidation.

## Glycerophospholipids in ALS

Glycerophospholipids are major structural components of all eukaryotic plasma membranes [50, 51]. Phospholipids form the characteristic phospholipid bilayer, and their composition affects membrane geometry, fluidity, and permeability [50, 51]. Some species also function as bioactive signaling molecules. A majority of the brain’s arachidonic acid and DHA are stored in the membranes as PE and PS [117].

PC is a main source of acetylcholine in the CNS, and intake of PC can improve memory and learning and ameliorate cognitive decline in mouse models of dementia [118–120]. Elevated levels of PC have been reported in the CSF [65] and the spinal cord nuclear lipidome [70] of ALS patients, the spinal cords of FUS overexpression mice [68], the skeletal muscle of SOD1-G86R mice [67], and the motor cortex of SOD1-G93A mice [69]. Elevated levels of PC 44:8 and PC 36:4 have been found to be the most discriminatory in the CSF and plasma, being able to differentiate between slow- and fast-progression cases [61, 65]. Elevated levels of PC 36:4 are also observed in SOD1-G93A mouse brains [66].

In a longitudinal study of ALS patients across two years, several species of PC and PS were decreased in patient blood in the initial stage of pathogenesis, with reductions in PS 38:1 and PS 40:7 being discriminatory even at baseline. Follow-up samples had elevated levels of these PCs, suggesting an increased level with disease progression. Despite an initial decline, PE levels increased progressively in the ALS patients and PE 40:7 could even be used to discriminate ALS from PLS [62].

Lysophospholipid levels are reported to be elevated in plasma [34, 60, 61, 63], CSF [65] and spinal cords [35, 70] of ALS patients, in the spinal cords of FUS-overexpression mice [68], and in skeletal muscles of SOD1-86R mice [67]. Lyso-PC 18:2 is commonly discriminatory in ALS patient CSF and SOD1-G93A mice brains, along with elevation of lysoPCs containing the long-chain fatty acids C16:0, C18:0 and C18:1 [65]. Specific species of lysoPC esters and lysoPE plasmalogens are significantly and progressively reduced in ALS patient blood samples [62]. A study testing for levels of lysoPCs containing fatty acids with various saturation status reported an increase of lysoPCs containing C16 and C18:1n9 fatty acids in spinal cords of both ALS patients and SOD1-G93A mice [35]. Lyso-PCs are generally a by-product of cholesterol ester synthesis that is elevated in ALS conditions. Addition of these lysoPC species (C16, C18, and C18:1n9) to motor neuron cultures in vitro causes motor neuron toxicity and death compared to their corresponding free fatty acids (C16, C18, C18:1) as controls, with lysoPC C16 being more toxic than others [35]. The data suggest that the elevated lysoPC level may be detrimental for motor neurons.

## Sphingolipids in ALS

Sphingolipids are a class of lipids which are ubiquitously found in cell membranes and are an integral constituent of lipid rafts, contributing to membrane stability and permeability [50, 121]. Sphingolipids are highly enriched in the CNS, where different CNS cell types have different sphingolipid profiles [122]. Due to this diverse distribution, sphingolipids have been shown to be vital in brain development, neurogenesis, differentiation, axonal growth and ageing [46, 123, 124]. Furthermore, sphingolipids are important biomarkers for neuroinflammation and several neurodegenerative diseases such as Alzheimer’s disease, Parkinson’s disease, multiple sclerosis and ALS [125–130]. Breakdown of sphingolipids takes place in the lysosome and defects in this process can lead to accumulation of sphingolipids, which is implicated in many neurological diseases, including ALS. Mutations in enzymes catalyzing the degradation of these sphingolipids are responsible for a large group of lysosomal storage diseases, also called sphingolipidosis [131], which is often manifested as ALS-like symptoms (see below). Collectively, these observations suggest that homeostatic regulation of sphingolipid metabolism is essential for CNS function (Fig. 2).

### Ceramides

Ceramides consist of a sphingosine attached to a fatty acid tail, and are the precursors to the more complex sphingolipids (Fig. 3). They are primarily generated by de novo synthesis, and from the breakdown of more complex sphingolipids, especially the breakdown of SM. The first and rate-limiting step of de novo ceramide synthesis is the condensation of palmitoyl-CoA and *L*-serine, catalyzed by SPT, to form 3-keto-sphinganine, which is reduced to sphinganine, a key intermediary. Sphinganine is *N*-acylated by one of the ceramide synthases (CerS), each of which has a preferential specificity for fatty acyl CoAs of different carbon-chain lengths, leading to the formation of dihydroceramides with different chain lengths, which are then desaturated to form ceramides [45, 132, 133].

An early milestone study on the role of sphingolipids in ALS was published in 2002 [33]. Cutler and colleagues quantified various lipids in ALS patient spinal cords, and found accumulations of ceramides, SM, and cholesterol esters along with increased oxidative stress. Similar results were obtained at the pre-symptomatic stage in the spinal cords of SOD1-G93A mice. To understand the relationship among oxidative stress, deficits in sphingolipid biosynthesis and cell death, cultured motor neurons were treated with either DMNQ, an oxidative stress-inducing agent, or palmitoyl-CoA, the initial substrate for sphingolipid synthesis catalyzed by SPT. Either DMNQ or palmitoyl-CoA treatment alone increased the levels of ceramide and cholesterol esters within 6 h of exposure and triggered a dose-dependent cell-death. Combination of DMNQ and palmitoyl-coA resulted in exacerbated increase of ceramides, SM, cholesterol esters and cell death, all of which were reduced on treatment with DMNQ and an SPT inhibitor [33]. Thus, their data suggest that oxidative stress acts through enhanced sphingolipid synthesis to induce neuronal death. Furthermore, ceramide induces apoptotic cell death in cortical and motor neurons [134–137], suggesting that accumulation of ceramides could contribute to ALS pathogenesis [33]. Indeed, elevated levels of ceramide have been reported in spinal cords and motor cortex of SOD1-G93A rats [69], as well as in plasma [60, 62, 63], spinal cords [33, 36] and fibroblasts [73] of ALS patients. Furthermore, increased ceramide levels are observed in spinal motor neurons, but not in ocular motor neurons, derived from ALS patients [18]. Taken together, these data suggest that accumulation of ceramide, a precursor for sphingolipids, could contribute to ALS pathogenesis.

Recent whole-exome sequencing in juvenile- and adult-onset ALS patients identified several genetic variants of *SPTLC1* [16, 17]. SPTLC1 is the long-chain subunit 1 of the enzyme SPT, which is the rate-limiting enzyme for ceramide biosynthesis. At least two not-mutually-exclusive mechanisms have been proposed for these dominant-acting *SPTLC1* variants.

The first mechanism takes cue from hereditary sensory neuropathy type1 (HSAN1), a disease characterized by atrophy of sensory neurons [138, 139]. Mutations in *SPTLC1* are the underlying cause of HSAN1. In HSAN1, the *SPTLC1* variants cause alterations of substrate specificity of the SPT enzyme from *L*-serine to either *L*-alanine or *L*-glycine, leading to the formation of 1-deoxysphingolipids instead of ceramides. These atypical 1-deoxysphingolipids cannot be synthesized into more complex sphingolipids or degraded, thereby resulting in accumulation of atypical 1-deoxysphingolipids that are highly neurotoxic [140]. HSAN1 patients are often treated with oral supplementation of *L*-serine to reduce production of the toxic 1-deoxysphingolipids [141]. The p.S331Y *SPTLC1* variant [16] identified in juvenile ALS has been previously reported as an atypical HSAN1 variant with a distinct mixed sensorimotor neuropathy phenotype [142]. Furthermore, using cell culture assays, Johnson et al*.* reported that the p.A20S *SPTLC1* variant also showed an altered substrate preference for *L*-alanine and *L*-glycine, along with mitochondrial defects, which were rescued on exposure to *L*-serine [16]. Thus, these observations indicate that the ALS-linked variants alter substrate specificity of SPT [142, 143].

The other juvenile ALS variants identified are distinct from HSAN1 variants and map to exon 2 of *SPTLC1,* including p.A20S, p.Y23F, p.L39del and p.F40_S41del*.* This exon codes for a transmembrane domain that interacts with ORMDL proteins to inhibit SPT activity [17, 144]. Mohassel et al*.* showed that these juvenile ALS variants are not sensitive to ORMDL protein levels, resulting in higher levels of sphinganine and ceramides. Correspondingly, increased levels of ceramides, but not 1-deoxysphingolipid (a HSAN1 characteristic feature), are found in the sera of juvenile ALS patients with variants p.A20S, p.Y23F, p.L39del and p.F40_S41del [17]. It should be noted that Mohassel et al*.* did not test substrate specificity preferences of the variants. Furthermore, selective knockdown of the ALS *SPTLC1* allele restored normal ceramide levels in human iPSC motor neurons [17]. Thus, these ALS-linked variants of *SPTLC1* could disrupt regulation of SPT, resulting in unrestrained activity and higher ceramide levels.

All the evidence presented above indicates that increased accumulation of ceramides is a common theme for ALS. Thus, prevention of ceramide accumulation could be a potential therapeutic intervention and can be achieved by promoting its synthesis to other sphingolipids or its degradation. Indeed, inhibition of glucosylceramide synthase (GCS), the enzyme which synthesizes glucosylceramide from ceramides, accelerates disease progression in SOD1-G93A mice [36]. Genetic deficiency of ceramidase, the enzyme which degrades ceramides, is causal to Farber disease, a lipid storage disease with some patients exhibiting muscle weakness and seizures [145]. Probing the potentials of ceramides as a therapeutic target is further discussed in “Potential therapeutic interventions targeting lipids” section.

### Sphingomyelin

As the name suggests, SMs are abundantly present in the myelin sheath. They are the most abundant sphingolipid found in cell membranes and play a critical role in maintaining myelin sheath integrity and function and in neuroinflammation and signal transduction [51, 146–149]. Sphingomyelinase (encoded by *SMPD1*) breaks down SM into ceramide and phosphocholine. Mutations in *SMPD1* cause accumulation of SM in the CNS, leading to dementia, ataxia, and slurred speech as seen in Niemann-Pick disease type A and B [150–152] (Fig. 3).

SM accumulation has been reported in plasma [34, 60, 61, 63], CSF [65], spinal cords [33, 36] and fibroblasts [73] of ALS patients. Elevated levels of SM 22:3, SM 24:1, SM OH22:2, and SM 16:1 in the plasma are found to be accurate discriminators of ALS disease progression and predictors of clinical indicators in a study with 74 ALS patients [153]. Area-Gomez et al*.* additionally reported significantly lower levels of SM 20:0, SM 22:1 and SM 20:1 species in serum samples from ALS patients [62]. The expression profiling of SMs in ALS mouse models remains inconclusive. Cutler et al*.* reported an increase of SM in the spinal cords of SOD1-G93A mice from pre-symptomatic to post-symptomatic stage [33]. By contrast, Dodge et al*.* reported an initial decrease of SM at paralysis onset, but a normal sphingomyelin level at full-paralysis stage in the spinal cords of SOD1-G93A mice [36]. The expression levels of SM are mixed in SOD1-G86R mouse spinal cord and skeletal muscle [67], and are reported to be lower in mice overexpressing wild-type human FUS [68].

### Glucosylceramides

Glucosylceramides are the essential first step to the synthesis of gangliosides, a major component of neurons. As such, glucosylceramides are vital for brain development. Glucosylceramide is synthesized from ceramides by GCS and broken down into ceramides by glucocerebrosidase-1 (GBA1) and GBA2 (Fig. 3). Mutations in GBA1 lead to Gaucher disease, which is characterized by neurological symptoms such dementia and ataxia, and treatment strategies include administration of recombinant human GBA [154]. Furthermore, GBA variants are risk factors for several neurodegenerative diseases, such as Parkinson’s disease, hereditary spastic paraplegia, and spinocerebellar ataxia [155–157]. While mice with neuronal specific knockout of GCS are born with severe neural defects [158], inhibition of GCS activity extends the survival of mouse models of Gaucher disease [159]. Thus, the balance between GCS and GBA activity in maintaining glucosylceramide levels in the CNS is critical for brain health, and an imbalance may lead to neurodegenerative conditions.

Elevated levels of glucosylceramide have been observed in plasma [60], CSF [65] and spinal cords [36] of ALS patients, spinal cords of SOD1-G93A mice [36], motor cortex of SOD1-G93A rats [69], and skeletal muscles of SOD1-G86R mice [67]. GCS activity is reported to be upregulated in skeletal muscles of SOD1-86R mice and ALS patients [67], and in the spinal cords of ALS patients and SOD1-93A mice [36]. However, unlike that in Gaucher disease, inhibition of GCS activity causes a loss of motor strength and neuromuscular junction integrity in wild-type mice [67], and significantly speeds up disease progression in SOD1-G93A mice [36]. Thus, the data suggest that glucosylceramide accumulation plays a neuroprotective role in ALS. Indeed, conduritol B epoxide inhibition of GBA that breaks down glucosylceramide, improves NMJ integrity, increases ganglioside GM1a and slows disease progression in SOD1-86R mice [37]. The conduritol B epoxide treatment also increases recovery from sciatic nerve injury in wild-type mice [37]. Similar alleviation of disease symptoms and improved survival are reported in SOD1-86R mice when they are treated with ambroxol hydrochloride, a GBA inhibitor [38].

### Galactosylceramides and lactosylceramides

Galactosylceramides are reported to be depleted in ALS patient blood samples [61], but elevated in spinal cord samples of patients [36]. The enzymatic activities of galactocerebrosidase and galactosylceramide synthase involved in regulating galactosylceramide levels, are also elevated in spinal cords of SOD1-G93R mice [36]. Lactosylceramides are synthesized from glucosylceramides and have been reported to be elevated in blood [60–62], spinal cords [36] and nuclei [70] of ALS patients. In addition, galactocerebrosidase mutations are causal for Krabbe disease [160], another motor degenerative disease, while lactosylceramides are activators of neuroinflammation [161]. Further studies are needed to explore the role of these ceramides in ALS,

### Gangliosides

Gangliosides with complex carbohydrate head groups, are most abundantly found in the CNS and are involved in several functions such as cell–cell recognition, signal transduction, synaptic  transmission, cognition and oligodendrocyte differentiation [162, 163]. The composition of gangliosides within the CNS changes during neurodevelopment: from simplest gangliosides GD3 and GM3 expressed primarily in early development stages, to more complex gangliosides such as GM1, GD1a and GD1b dominate in later stages and adult brains [164, 165]. Lack of ganglioside synthesis can cause epileptic seizures and neurodegeneration, while changes in ganglioside levels and classes have been associated with various neurodegenerative diseases such as Huntington’s disease, Alzheimer’s disease and Parkinson’s disease [46, 128, 166–168]. Accumulation of gangliosides causes lipid storage disorders called gangliosidosis. Intriguingly, gangliosidosis, such as Tay-Sachs disease, Sandhoff disease and GM1 gangliosidosis, often has clinical manifestations that mimic ALS [145, 169–171]. Both Tay-Sachs and Sandhoff diseases are caused by mutations in β-hexosaminidase subunits (HEXA and HEXB, respectively) that are required to breakdown GM2 to GM3, the latter of which is involved in neuronal growth, plasticity and repair [171, 172]. Therapeutic strategies for these diseases focus on preventing the buildup of GM2 in neurons, failure of which leads to toxicity, neuronal degeneration and eventual death [171, 173, 174].

Abnormal ganglioside composition in ALS has been reported in as early as 1980’s, along with reports of additional complex and unusual gangliosides found in ALS patient brains, spinal cords and CSF [175–177]. Further discovery of the presence of ganglioside antibodies in sera of ALS patients [178–180] fueled several clinical trials in the 1980’s. Unfortunately, these trials using exogenous bovine ganglioside for treatment yielded inconclusive improvement and results [181–183]. Subsequent studies provided more details on specific ganglioside profiles and dysregulation. In 2015, Dodge et al*.* reported increased levels of GM3 and GM1, along with increased activity of HEX in the spinal cords of ALS patients and SOD1-G93A mice [36]. Further, they showed that although increasing the HEX activity via adenoviral vector delivery to the CNS did not have any effect, direct intracerebroventricular delivery of GM3 significantly delayed the onset of paralysis and extended survival of SOD1-G93A mice. GM1 has been shown to amplify neurotrophic response, block excitotoxicity and promote neurite growth in rat models [184–186]. These findings suggest that the accumulation of GM1 and GM3 may be protective in nature and could be used to slow down ALS disease progression. The elevation of HEX expression has been observed in SOD1-G93A spinal astrocytes, which is associated with increased lysosomal and phagocytic activity [187].

In the same year (2015), Xu et al*.* showed that a dose of rHIgM12, a human antibody with binding specificity to the neuronal membrane gangliosides GD1a and GT1b, is able to delay disease onset and improve survival in both SOD1-G93A and SOD1-G86R ALS mouse models [188]. Both GD1a and GT1b are neuronal surface ligands for myelin-associated glycoprotein (MAG), binding of which inhibits nerve regeneration via membrane domain rearrangement. In culture, the MAG-mediated neurite growth inhibition is reduced by blocking ganglioside synthesis, modifying structure of the neural surface gangliosides or using antibodies against the gangliosides [189]. The data suggest that reducing the levels of GD1a and GT1b gangliosides or blocking their interaction with MAG may be beneficial.

## Sterol lipids in ALS

The major forms of sterols found in mammalian cells are cholesterol and its derivatives, such as oxysterols and cholesterol esters [40–42]. Cholesterol regulates membrane order and flexibility, is a component of membrane lipid rafts, and serves as a precursor to steroid hormones. In the CNS, cholesterol is implicated in synaptic formation, axonal growth, signal transduction, as well as learning and memory [190, 191]. Elevated levels of cholesterol in the sera of ALS patients are found to be discriminatory and prognostic for longer survival [22, 34]. Various cohort studies have shown a causal association of higher serum LDL-cholesterol with higher risk of ALS diagnosis [192–194]. However, post ALS diagnosis studies provided conflicting results on the levels of serum LDL, HDL and total cholesterol [21, 195–199]. Cholesterol levels are found to be elevated in the CSF of ALS patients [200]. Downregulation of the cholesterol metabolism pathway has been reported in a meta-analysis of transcriptomics studies in SOD1-G93A  mouse spinal cords [201]. Recently, two independent studies indicate that TDP-43, the key pathological hallmark protein for ALS [202], regulates SREBF2-mediated cholesterol metabolism [28, 29]. Furthermore, the expression of 3-hydroxy-3-methylglutaryl-CoA reductase (HMGCR), a rate-limiting enzyme for cholesterol biosynthesis and a transcription target of SREBF2, is reduced in oligodendrocytes bearing TDP-43 pathologies [28], suggesting that cholesterol metabolism may be affected in cells with TDP-43 proteinopathies. Although no change is observed in free cholesterol levels in the sera of ALS patients [29] as well as spinal cords of ALS patients and SOD1-G93A mice [35], the cholesterol level is reduced in the CSF of ALS patients [29]. Furthermore, reduced levels of lanosterol, a precursor to cholesterol, are observed in ALS patients and SOD1 mouse models, along with downregulation of HMGCR [35].

Given that cholesterol cannot cross the blood–brain barrier, cholesterol is synthesized and stored in the CNS without peripheral contribution [203, 204]. It is questionable if peripheral serum levels of cholesterol reflect the levels in the CNS, and vice versa. This disparity may explain why studies using statins to reduce serum cholesterol in ALS patients showed no effect or negative effect on disease progression [205, 206]. There is a large discussion surrounding the interplay of cholesterol metabolism, transport and uptake in the periphery and the CNS, and their effects in ALS. Please refer to Hartmann et al*.* 2021 for an in-depth discussion and summary of cholesterol metabolism studies in ALS [207].

Cholesterol cannot be degraded, and free cholesterol is toxic to the system. Excess cholesterol in the CNS is oxidized to oxysterols, which are able to cross the blood–brain barrier [208], and the blood levels of oxysterols are considered reflective of CNS status. The main forms of oxysterols found in the CNS are 24S-hydroxycholesterol (24-OHC), 25-hydroxycholesterol (25-OHC), and 27-hydroxycholesterol (27-OHC), of which 24-OHC and 27-OHC are LXR receptor ligands. LXR receptors activate expression of genes involved in cholesterol efflux pathway, such as ATP-binding cassette subfamily A member 1 (ABCA1) and APOE, thereby providing another layer to maintain cholesterol homeostasis. 25-OHC is found to be highly elevated in both serum and CSF of ALS patients, and is associated with disease severity and progression [198]. Levels of enzymes converting cholesterol into 25-OHC are found elevated in early symptomatic SOD1-G93A mouse brains [198, 201]. Additionally, 25-OHC induces neuronal death via LXR-mediated apoptosis in motor neuron-like cells containing the SOD1-G93A mutation [198]. Elevated levels of 24-OHC are found in spinal cords of ALS patients, and cause cell death in neuroblastoma cell lines [209]. These studies suggest a neurotoxic effect of accumulation of 24-OHC and 25-OHC in the CNS. GWAS studies have identified *CYP27A1*, which encodes the enzyme converting cholesterol to 27-OHC, as a susceptible loci for ALS [210]. However, lower levels of 27-OHC have been reported in the sera of ALS patients [199, 200], but show no correlation with survival [199].

Surplus free cholesterol can be esterified with fatty acyls to neutral cholesterol esters and stored in lipid droplets. In the CNS, this function is likely to be carried out primarily in astrocytes [47, 211]. Elevated cholesterol ester levels have been consistently reported in various tissues of ALS patients and animal models. Several species of cholesterol esters, including those with C16 and C18 saturated and unsaturated fatty acid chains, are reported to increase by up to 22 folds in patient spinal cords, with a more pronounced effect in the grey matter [33, 35]. A four-fold progressive increase of C18 cholesterol ester species has been observed in SOD1-G93A mouse spinal cords from early symptomatic stage to end paralysis stage. Cholesterol esters levels are elevated in plasma samples and the change is maintained longitudinally, with increases in long- and very long-chain cholesterol esters, including CE 24:2 and CE 25:2 being discriminatory for ALS [62]. The SOD1-93A rat spinal cords have a sixfold increase of total cholesterol esters, mainly from PUFA species, including arachidonic acid (20:4). The cholesterol ester accumulation seen in the FUS-overexpressing mice is partially rescued on HDAC inhibition [68]. Mice with adenoviral-induced overexpression of SREBP2 transcription domain show motor neuron degeneration, paralysis and reduced survival accompanied by accumulation of cholesterol esters [35]. Interestingly, lysoPC, a by-product of cholesterol ester synthesis, is also elevated in spinal cords of both patients and SOD1-G93A mice [35]. Lyso-PC causes rapid demyelination and is shown to be toxic to motor neuron cultures [35], suggesting that accumulated cholesterol esters may be toxic via action of their by-product.

## Potential therapeutic interventions targeting lipids

**Fig. 4:**
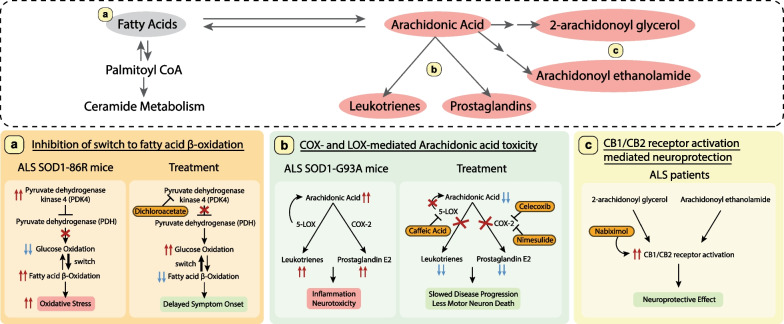
Potential therapeutic strategies targeting fatty acids. Upper panel shows an overview of fatty acid metabolism intervention points with neuroprotective effects. Fatty acids and derivatives shown to be toxic in ALS are highlighted in salmon pink. Lower panels describe the toxicity and the therapeutic strategies used in ALS mice and ALS patients. **a** Inhibition of the switch to fatty acid β-oxidation, **b** COX- and LOX-mediated arachidonic acid toxicity, and **c** CB1/CB2 receptor activation-mediated neuroprotection. Intervening compounds are in orange

As discussed above, it is apparent that lipid dysregulation, in particular accumulation of toxic lipid species, contributes to ALS. As such, targeting these toxic lipids makes for attractive therapeutic interventions. Indeed, various strategies have shown to be successful in alleviating disease symptoms, extending survival and providing neuroprotection in vitro and in animal models. In this section, we present an overview of lipids as therapeutic targets for ALS treatment (Fig. 3, 4).

### Inhibition of the switch from glucose oxidation to fatty acid β-oxidation

Glucose oxidation is the main energy source in the brain, while fatty acid β-oxidation contributes to up to one-fifth of the total brain energy needs [77]. Although fatty acid oxidation produces more ATP as compared to glucose, it also takes up more oxygen resources. As such, cells with prolonged fatty acid β-oxidation undergo oxidative stress, thereby producing harmful reactive-oxygen species. Due to the high energy demand and impaired glucose metabolism in ALS [24, 212], there is a switch to fatty acid β-oxidation as the major route for energy generation, placing the system under increased oxidative stress, a key mechanism of neurotoxicity in ALS [23, 79, 80]. It is worth noting that the switch to the use of fatty acids as an energy source has been observed in skeletal muscles of SOD1-G86R [79] and SOD1-G93A mice [213] even prior to disease onset.

Pyruvate and fatty acyl CoA are important intermediates of glucose and fatty acid oxidation, which are converted to acetyl-coA. The acetyl-coA enters the TCA cycle to generate ATP. Pyruvate dehydrogenase catalyzes the oxidation of pyruvate to acetyl-coA and is inhibited by pyruvate dehydrogenase kinase 4 (PDK4) to regulate pyruvate levels. Palamiuc and colleagues demonstrated that *Pdk4* expression is elevated in skeletal muscles of SOD1-86R mice accompanied by impaired glucose metabolism and a switch to fatty acid β-oxidation, leading to greater oxidative stress [79]. Also, *Pdk4* mRNA expression was found with a three-fold elevation in ALS patient muscles [79]. Inhibition of PDK4 with dichloroacetate in the SOD1-86R mice leads to a switch back to increased glucose oxidation, and the mice show improved mitochondrial function, reduced muscle denervation, and delayed disease onset [79]. These results underscore the importance of metabolic switch in inducing oxidative stress and disease pathogenesis (Fig. 4a).

### Inhibition of COX- and LOX-mediated arachidonic acid toxicity

Elevated levels of arachidonic acid and its derivatives have been reported in ALS patients and models [18, 34, 60, 103–107]. Arachidonic acid produces prostaglandins and leukotrienes via the COX and LOX pathways. These molecules can induce inflammation and cause motor neuron death, which could be rescued by treatment with LOX and COX inhibitors. Administration of nimesulide, an inhibitor for COX-2, also shows great promise, as it reduces the level of PGE_2_ in the spinal cord and delays disease onset in SOD1-G93A mice [214]. Administration of 5-LOX inhibitor caffeic acid, apigenin or nordihydroguaretic acid promotes survival of ALS spinal motor neurons in vitro, reverses eye degenerative phenotypes and promotes survival in C9orf72 ALS flies [18]. Direct treatment with arachidonic acid increases cell death of ALS spinal motor neuron cell lines, which can be rescued in a dose-dependent manner by caffeic acid [18]. In SOD1-G93A mice, caffeic acid reduces astrocyte and microglia activation, maintains neuromuscular junction morphology and architecture, delays disease onset and prolongs lifespan [18] (Fig. 4b). 5-LOX-mediated toxicity is a well-established mechanism in many types of cancer and several inhibitors including caffeic acid have been tested and are under clinical trials as chemotherapeutic agents [215, 216]. It would be of interest to make use of these studied chemotherapeutic agents as candidates for ALS therapeutics.

PGE_2_ is a key mediator in the initiation of inflammatory oxidation and propagation. Inhibition of COX-2, an enzyme involved in PGE_2_ synthesis, and downregulation of PGE_2_ receptor, have been shown to delay the onset of ALS symptoms in SOD1-G93A mice [214, 217]. Given the involvement of PGE_2_ in ALS inflammation and potential systemic side effects of COX-1 inhibition (COX-1 is constitutively expressed in most tissues), a variety of COX-2-inhibiting non-steroidal anti-inflammatory drugs (NSAIDs) have been tested for ALS therapeutics. Administration of NSAIDs, such as rofecoxib [218], nimesulide [214], and celecoxib [219], has been shown to delay disease onset and promote survival at varying degrees in SOD1-G93A mice [103, 105, 106] (Fig. 4b). Furthermore, depletion of TDP-43 in microglia, but not in astrocytes, increases COX-2 and PGE_2_ levels and reduces neural survival in vitro. This neurotoxicity could be rescued by celecoxib [220]. However, a double-blind clinical trial of celecoxib in 300 ALS patients showed no beneficial effects on muscle strength scored via the ALSFRS-R over a period of one year [221]. Although cohort studies to test for ALS risk associated with NSAID usage have been inconclusive [222, 223], a population study with 729 ALS patients found that the use of aspirin (a NSAID inhibiting both Cox-1 and Cox-2) may reduce ALS risk in people over 55 years [224]. Altogether, these studies indicate that activation of the arachidonic acid pathway contributes to ALS pathogenesis, and conversely, pharmacologic inhibition of the arachidonic acid pathway may have a therapeutic potential.

### Endocannabinoid-mediated therapies

SOD1-G93A mice treated with various cannabinoids with differing specificity to CB1 and CB2 receptors, like Δ^−9^THC [225], Sativex® [226], WIN55, 212–2 [112] and AM-1241 [227], show varied levels of disease progression delay and survival extension (Fig. 4c). There are few clinical trials testing for the effects of cannabinoids in ALS, and the number of patients employed was limited, with most early ones being observational (survey-based). Two clinical trials on the effect of tetrahydrocannabinol (Δ^−9^THC) in 27 and 9 ALS patients, respectively, showed no benefits [228, 229]. Another phase-2 clinical trial with 59 ALS patients tested nabiximols, an established drug used to treat muscle spasticity in multiple sclerosis [230]. The trial found that nabiximols is safe for use and has positive effects on muscle spasticity in ALS [230], opening avenues for large-scale clinical trials for ALS symptomatic relief. There is an ongoing placebo-controlled double-blind clinical trial with 30 ALS patients to study the efficacy of cannabis-based medicine extracts in slowing ALS progression as measured by the ALSFRS-R, and to evaluate its safety and effects in relieving pain and spasticity as well as improving quality of life [231]. It should be noted that 2-AG is also a substrate for COX-2, and it can be oxygenated by COX-2 to form various prostamides and prostaglandin glyceryl esters [232], such as Prostaglandin E2 glyceryl ester (PGE_2_-G) and prostaglandin D2 glyceryl ester with divergent physiological functions [233]. However, the role of these prostaglandin glyceryl esters in ALS remains to be explored and further studies are needed to explore the translation potential of endocannabinoids.

### Sphingolipid-related therapeutics

**Fig. 5:**
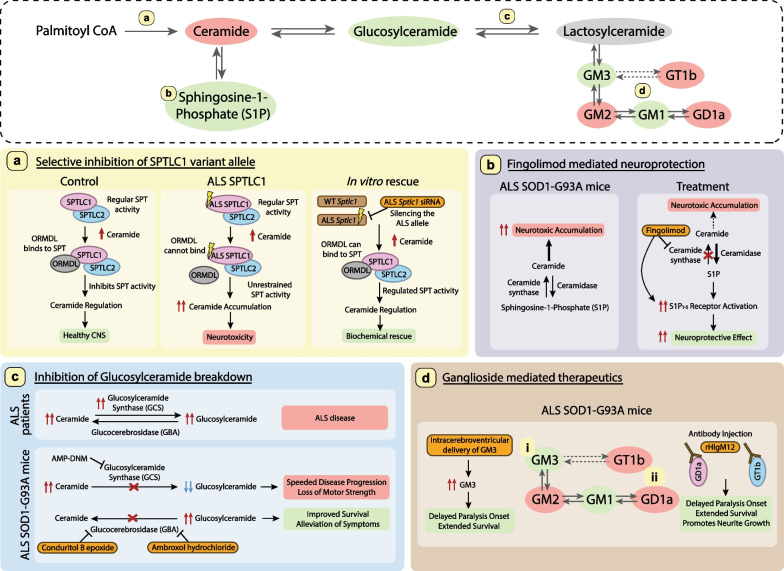
Ceramides and gangliosides therapeutic strategies. Upper panel shows an overview of sphingolipid metabolism intervention points. Sphingolipids with neurotoxic effects and neuroprotective effects in ALS conditions are highlighted in salmon pink and green, respectively. Lower panels describe the toxicity and the therapeutic strategies used. **a** Selective inhibition of *SPTCL1* variant allele, **b** fingolimod-mediated neuroprotection, **c** inhibition of glucosylceramide breakdown, and **d** ganglioside-mediated therapeutics. Intervening compounds, RNAs and antibodies are highlighted in orange

Given ceramide accumulation in patient tissues [33, 36, 60, 62, 63, 73] and the neuronal toxicity of ceramides [134–137], ceramides are both attractive biomarkers and therapeutic targets for ALS. Ceramide accumulation can occur through excessive synthesis, impaired degradation, and increased breakdown of more complex sphingolipids into ceramides (Fig. 5a–c). The recent identification of *SPTLC1* mutations [16, 17] further underscores this working model. ALS-linked mutations in *SPTLC1* cause altered substrate specificity and/or unrestrained activity of SPT (a key enzyme in ceramide synthesis), leading to accumulation of 1-deoxysphingolipids [142] or ceramides [17]. Selective knockdown of *SPTLC1* mutant allele using siRNA alleviates ceramide levels in vitro [17]*,* and the approach could possibly be extended in a clinical setting to revert accumulation of toxic lipids due to *SPTLC1* mutations (Fig. 5a).

Ceramides are also formed from the breakdown of glucosylceramides by hydrolyzing enzymes called glucocerebrosidases. Unlike in Gaucher disease, where glucosylceramide buildup causes liver and spleen malfunction [234], elevated levels of glucosylceramides in ALS models seem to play a neuroprotective role [36–38]. Inhibition of glucosylceramide synthesis accelerates disease progression [36, 67], while inhibition of glucosylceramide degradation via glucocerebrosidases, by conduritol B epoxide [37] and ambroxol hydrochloride [38], alleviates disease symptoms in mouse models, making glucosylceramides an attractive drug target to alleviate disease symptoms (Fig. 5c).

Additionally, mutations in ganglioside degradation enzymes can lead to lysosomal storage diseases and are the underlying cause of various neurodegenerative diseases such as Huntington’s disease, Alzheimer’s disease and Parkinson’s disease [166–168]. Multiple ganglioside species are involved in ALS: elevated levels of GM1 and GM3 are neuroprotective in nature [36, 184–186], while G1a and GT1b are toxic [189], and their inhibition by specific antibodies results in improved survival in ALS mice [188] (Fig. 5d). This divergence of roles within even a sub-class of lipids may explain the failure of early clinical trials that administered a broad array of bovine gangliosides for ALS treatment [181–183] without specificity, underscoring the importance of going in-depth of each molecule’s role.

### Other potential candidates

Ceramide degradation is catalyzed by ceramidases, and forms S1P. S1P is a bioactive lipid involved in regulation of many processes such as cell proliferation, survival, neuronal excitability, neuroinflammation and immune cell trafficking [127, 235, 236]. Fingolimod (FTY720 or Gilenya) is an agonist for S1P receptor and was approved as the first oral therapy drug for multiple sclerosis by the USA Food & Drug Administration in 2010 as it reduces relapse rate and brain volume loss and slows disease progression [237]. FTY720 is also a ceramide synthase inhibitor [238, 239]. It inhibits proinflammatory cytokine production and reduces T cell migration into the CNS, thus promoting neuroprotective role of microglia and preventing neuronal excitotoxicity [240–242]. FTY720 administration in SOD1-G93A mice prolongs survival, ameliorates neurological defects and regulates neuroinflammatory genes [39]. (Fig. 5b). A randomized double-blind phase IIa clinical trial of FTY720 has demonstrated short-term safety (4-weeks) of FTY720 with no adverse effects, reduction of circulating lymphocytes, and tolerability, suggesting the suitability for further clinical trials [243].

Other attractive candidate lipid biomarkers of therapeutic interest include cholesterol esters and lysoPC. Accumulation of cholesterol esters and particularly lysoPCs, has been consistently reported in ALS patient tissues and models [33–35, 60, 62, 63, 65, 70], and has been found to be discriminant for ALS [62, 65], with C16 and C18:1n9 lysoPC species commonly elevated in both patients and animal models [35]. Although synthesis of cholesterol esters protects cells from free cholesterol toxicity, the by-product of its synthesis, lysoPC, has been shown to cause rapid demyelination [244] and motor neuron death [35]. Lyso-PC species with C16 chains are highly neurotoxic and could be attractive targets for therapeutics.

Additionally, lower levels of TG in the CSF are associated with better survival [65]. This could be a result of their breakdown to fatty acids to effectively meet energy demands in disease conditions. Additionally, although not demonstrated in the ALS context, neurotoxic reactive astrocytes in vitro increasingly secret long-chain saturated fatty acids which were shown to be neurotoxic [88]. Therefore, oxidative stress may result in neurotoxic reactive astrocytes in ALS [28, 245], which could be another potential mechanism to explore for a better understanding of ALS pathogenesis.

## Open questions

There are of course many outstanding questions in the field to consider especially in deciphering the mechanisms underlying lipid toxicity or protection in ALS. Two areas of interest we would like to highlight are the interaction between CNS and peripheral lipid levels, and the cell-type specific lipid metabolism. The blood–brain barrier only allows selective molecules to pass freely between the CNS and the periphery, making it important to study lipid distribution in various tissues to better understand lipid synthesis, mobilization, uptake and consumption. Various tissues from ALS patients like blood, CSF, spinal cord and skeletal muscle have distinct lipid profiles (summarized in “Overview of ALS lipidomic studies” section), with no common overlapping differential species even between blood and CSF of the same ALS patients [61]. These distinct profiles are highlighted in the context of TGs, which can pass through the BBB, and cholesterols, which cannot cross the BBB. TG levels are elevated in blood [61–63] and depleted in the CSF [61, 65], which may be a result of mobilization of TGs from the peripheral adipose tissue to meet greater energy demands and consumption of TGs in the CNS in diseased state. Cholesterol levels are reported to be elevated in the plasma [34, 62], which may be a risk factor for ALS in the pre-diagnostic stage [192], but they are downregulated in mouse spinal cords. In fact, studies using statins, which cannot cross the BBB, to control blood cholesterol levels have failed [205, 206], raising the question of the diagnostic or therapeutic value of blood cholesterol levels, and emphasizing the need for a better understanding of the exchange of lipids between the CNS and the periphery.

A recent lipidomic study using primary cells (i.e., cortical neurons, astrocytes, and oligodendrocytes) revealed that each CNS cell type has its unique lipid-class signature and cell-type specific lipid species [81]. For example, levels of cholesterol and ceramide are highest in neurons; astrocytes are enriched with PS, phosphatidylinositol and diacylglycerol; sulfatide and hexosylceramide are enriched in oligodendrocytes; and levels of SM and phosphatidylglycerol are highest in microglia [81]. These cell-type specific signatures may reflect the specialized functions of each cell type. Furthermore, lipid biosynthesis may be developmentally regulated and context-dependent. For example, cholesterol biosynthesis is required in neuroprogenitor cells but dispensable in mature neurons [246]. During myelination, oligodendrocytes upregulate its own cholesterol biosynthesis, as well as taking up cholesterol synthesized in astrocytes [247, 248]. Further work is needed to understand the cell-intrinsic mechanisms underlying the processing and functions of these lipids as well as how lipid dynamics may be regulated via cell–cell communication.

In the CNS, astrocytes are the main site for lipid oxidation and storage [78]. During high energy demand, the lipids synthesized in neurons are transported into astrocytes, forming lipid droplets, and undergoing oxidation [78]. Astrocytes contain a greater number of antioxidant molecules and help consume the damaging reactive oxygen species produced from lipid oxidation. A recent study demonstrated that astrocytes become reactive in response to oxidative stress, secreting long-chain saturated fatty acids which are neurotoxic [88]. Furthermore, we and others have shown that TDP-43-depleted astrocytes and ALS astrocytes may shift towards the inflammatory reactive state [249, 250]. Another study using ALS spinal cord motor neuron cultures showed elevated levels of arachidonic acid, which are autonomously neurotoxic [18]. Whether ALS astrocytes may exacerbate the lipid-mediated toxicity toward ALS motor neurons is an intriguing possibility and remains to be tested. Moreover, we have shown that depletion of TDP-43 in oligodendrocytes alone is able to produce motor deficits via SREBP2-mediated cholesterol downregulation [28]. Evidence of TDP-43-mediated cholesterol dysregulations has been found in oligodendrocytes harboring TDP-43 pathologies [28]. Furthermore, CSF cholesterol level is reduced in ALS patients [29]. These studies together suggest complex cell–cell communication and transport, and perhaps a cell-type specific mechanism of lipid-mediated toxicity in ALS. How the lipid dynamics change during normal and disease conditions remains to be addressed.

While this review focuses on lipids, it should be noted that lipid species or various metabolites are interconnected. For example, it has been recently shown that synthesis of highly unsaturated fatty acids could provide nicotinamide adenine dinucleotide (NAD^+^) production as a homeostatic regulation for maintaining REDOX homeostasis [251]. In this regard, leveraging on NAD^+^ metabolism by either supplying NAD^+^ precursors or inhibiting NAD^+^-consuming enzymes has been proposed as an option for ALS treatment [252, 253]. In addition, a recent study has also highlighted that gut microbiome and metabolites may modulate ALS pathogenesis [254]. How these metabolites affect each other, and what serve the initiating factors would be of great interest to be resolved.

## Conclusions

The great structural diversity of lipids allows for diverse functions, and thus diverse and complex roles in various mechanisms of ALS pathogenesis. Given their assorted roles, the question of whether lipidemia dysregulation is the cause or the consequence of the disease is not easily answered. Lipid changes could be both causal and a consequence of disease pathology, forming complex feedback and feedforward regulations. ALS-linked mutations in *SPTLC1* [16, 17] affect activity and/or substrate specificity of SPT, leading to accumulation of toxic lipids. Elimination of *SPTLC1* mutants can reduce accumulation of putative toxic lipids [17], while reducing toxic lipids rescues cellular phenotypes [16], suggesting that lipid dysfunction could drive ALS pathogenesis. The prevalence of prognostic and pre-diagnostic dyslipidemia risk factors for ALS, such as blood cholesterol levels [192] and body mass index [255], as well as evidence showing that inhibiting the pre-diagnostic energy source switch to fatty acid oxidation alleviates disease symptoms in mice [79, 213], further support a causal role of lipids in ALS. By systematically assessing the current literature, we highlight that accumulation of ceramides, arachidonic acid, lysoPC, and cholesterol esters is emerging as a common theme that is detrimental to motor neurons. Conversely, reducing the accumulation of these toxic lipids, in particular, ceramides and arachidonic acid, appears to be beneficial in various ALS models. Furthermore, increased levels of potentially beneficial lipids such as glucosylceramides, and activation of S1P-mediated signaling may be protective in ALS. We look forward to future investigations to restore the faulty lipids in ALS.

## Data Availability

Not applicable.

## Abbreviations

2-AG: 2-Arachidonoyl glycerol
24-OHC: 24S-Hydroxycholesterol
25-OHC: 25-Hydroxycholesterol
27-OHC: 27-Hydroxycholesterol
AEA: Arachidonoyl ethanolamide, or anandamide
ALS: Amyotrophic lateral sclerosis
ALSFRS-R: ALS functional rating scale - revised
APOE: Apolipoprotein E
C9ORF72: Chromosome 9 open reading frame 72
CARM1: Coactivator-associated arginine methyltransferase 1
CB1: Cannabinoid receptor 1
CB2: Cannabinoid receptor 2
Cer: Ceramide
CerS: Ceramide synthase
CNS: Central nervous system
COX: Cyclooxygenases
cPLA2: Cytosolic phospholipase A2
CSF: Cerebrospinal fluid
DHA: Docahexaenoic acid
ELOVL1: ELOVL fatty acid elongase 1
EPA: Eicosapentaenoic acid
FUS: Fused in sarcoma
GBA1: Glucocerebrosidase-1
GBA2: Glucocerebrosidase-2
GCS: Glucosylceramide synthase
GWAS: Genome-wide association study
HDL: High-density lipoprotein
HEX: Beta-hexosaminidase
HEXA: Beta-hexosaminidase subunit alpha
HEXB: Beta-hexosaminidase subunit beta
HMGCR: 3-Hydroxy-3-methylglutaryl-CoA reductase
HSAN1: Hereditary sensory neuropathy type1
iPSC: Induced pluripotent stem cells
LDL: Low-density lipoprotein
LOX: Lipoxygenases
5-LOX: 5-Lipoxygenase
LXR: Liver X receptor
LysoPC: Lysophosphatidylcholine
LysoPE: Lysophosphatidylethanolamine
MAG: Myelin associated glycoprotein
MUFA: Monounsaturated fatty acids
NMJ: Neuro-muscular junction
NSAIDs: Non-steroidal anti-inflammatory drugs
ORMDL: Orosomucoid-like
PC: Phosphatidylcholine
PDK4: Pyruvate dehydrogenase kinase 4
PE: Phosphatidylethanolamine
PGE_2_: Prostaglandin E2
PLS: Primary lateral sclerosis
PS: Phosphatidylserine
PUFA: Polyunsaturated fatty acids
S1P: Sphingosine-1-phosphate
SCD: Stearoyl-CoA desaturase
SM: Sphingomyelin
SOD1: Superoxide dismutase 1
SPT: Serine palmitoyltransferase
SPTLC1: Serine palmitoyltransferase long chain base subunit 1
SREBP2: Sterol regulatory element-binding protein 2
TARDBP: TAR DNA binding protein
TDP-43: Transactive response DNA binding protein 43 kDa
TG: Triglyceride
